# Study of a Single Joint Between Two FDM-Printed PLA Filaments

**DOI:** 10.3390/polym17233106

**Published:** 2025-11-22

**Authors:** Jaime Orellana-Barrasa, Emilio Antón Carrasco-Otermín, José Ygnacio Pastor

**Affiliations:** Centro de Investigación en Materiales Estructurales (CIME), Departamento Ciencia de Materiales, Universidad Politécnica de Madrid, 28040 Madrid, Spain; emilio.carrasco@alumnos.upm.es (E.A.C.-O.); jy.pastor@upm.es (J.Y.P.)

**Keywords:** fused deposition modelling (FDM), polylactic acid (PLA), single joint characterization, cruciform sample, cross-shaped sample, printing temperatures

## Abstract

Isolating the mechanical properties of an FDM joint by performing a direct tensile test on it is something that has yet to be achieved. Developing a methodology for isolating the properties of a single joint could help to inform simulations and achieve a better understanding of the mechanisms affecting the bond strength between FDM-printed materials. In this work, a cruciform single-joint test (CSJT) of a cross-shaped specimen and a fast mechanical clamping protocol are introduced to evaluate the apparent tensile strength and fracture mechanisms of a single FDM-printed joint between two PLA filaments. First, a discussion of different approaches for obtaining a fast, reproducible, and reliable test of the samples is presented. Then, nozzle temperature (180–215 °C) and bed temperature (30–120 °C) were systematically varied, producing a minimum of *n* = 12 samples per condition. Samples were classified after failure, depending on the fracture mechanism (type 1 = joint failure; type 2 = filament failure), and the apparent tensile strength (ATS) of the joint was computed from the tensile tests and optical micrographs. The detachment probability of the joints decreased sharply above 210 °C, while the ATS increased, approaching a plateau near ~50 MPa. The influence of bed temperature was smoother, with a stable decrease in the detachment ratio as the ATS increased, indicating that nozzle temperature is the main factor contributing to the joint strength. These results map a temperature-driven transition from joint-controlled to filament-controlled failure. The method proposed also provides a minimal-material, high-throughput route to quantify FDM interlayer bonding and inform process simulations. Additional tests are performed to contextualize the results presented.

## 1. Introduction

Additive manufacturing (AM) comprises a family of processes that fabricate three-dimensional (3D) artefacts in a layer-wise process directly from a digital file. In contrast to conventional subtractive routes, which remove material to achieve the desired geometry, AM deposits or consolidates material only where required, thereby reducing waste and enabling near-net-shape production [1,2]. The AM field spans multiple processes, including stereolithography (SLA) [3,4], fused deposition modelling (FDM) [5], selective laser sintering (SLS) [6], material jetting (MJ) [7], binder jetting (BJ) [8], directed energy deposition (DED) [9], and electron beam powder bed fusion [10], each with distinct feedstocks, energy–matter interactions, and resolution–throughput trade-offs. Owing to its unprecedented geometric freedom (e.g., lattice and topology-optimized structures), customization, and rapid iteration, AM has been adopted across industries, evolving from rapid prototyping in its early days to the increasingly widespread production of certified end-use parts [11,12,13,14].

Additive manufacturing technologies rely fundamentally on the adhesion between layers of materials, a mechanism that directly affects the mechanical integrity and performance of the fabricated components [5,15,16]. The quality of interlayer bonding is crucial for the mechanical strength of the produced components, making their characterization a vital aspect for both research and industrial applications. Understanding the factors that influence layer adhesion, including processing parameters, material properties, and environmental conditions, is crucial for optimizing additive manufacturing processes and ensuring the reliable performance of final products.

Numerous studies have investigated the mechanical properties of FDM-printed PLA, with particular interest in those addressing the interlayer adhesion and fracture energy. These studies have employed various tests, including tensile tests [17,18,19,20,21], fatigue tests [22], bending tests [23], double cantilever beam tests [24], shear tests [25], and tearing tests [26]. These investigations, together with those evaluating the mechanical strength of FDM-printed samples, provide a broad characterization on the effect of different parameters: processing parameters (layer height [16,23,24,27,28,29,30], raster angle [29], nozzle temperature [23,24,31,32,33], bed temperature [23,24,31,32], printing speed [16,29,31,32,33,34,35], wait-time [17,23,32], nozzle diameter [30,35,36], feed rate [33], and heat treatments [18,20,34,37,38]), sample parameters (sample thickness [29,39], porosity or gaps [18,19,21,27,40], stress concentrators [19], filament orientation [19,27,39], infill patterns and densities [16,39,41]), or material parameters (crystallinity [18,20,37], road-bonding [20], thermal stress [18], filler type and content [31,37,39]).

This review of the literature, alongside deeper reviews published on the topic [5,15], manifests two things:

- There is excellent complexity in understanding the isolated effect of any variable on the interlayer strength. As discussed by Allum J. et al. [8], the literature assessing interlayer adhesion presents a variety of geometrical designs, which makes the comparison of results difficult and yields opposing conclusions for the effect of the same variable on interlayer strength.
- A lack of studies focusing exclusively on the characterization of a single, isolated, interlayer joint under controlled conditions. This specific approach is crucial for understanding the intrinsic properties of the interlayer bond by using the simplest possible joint that can be produced.

This study aims to address the gap in the literature and design experiments that provide deeper insights into the fundamental mechanisms governing interlayer adhesion. It will help inform the development of more robust additive manufacturing processes, as previously achieved in our group’s study of 1-D filaments [42,43,44,45], and provide new data for machine learning algorithms [42]. For this purpose, a cross-shaped or cruciform sample has been designed with two PLA filaments joined or welded at a single point. They have been printed using an FDM process, and the influence of two key processing parameters (the nozzle temperature and the bed temperature) has been evaluated. This study presents a discussion on the various approaches for testing a single PLA–PLA joint (adhesive clamping vs. mechanical clamping), evaluates their respective advantages and limitations, and proposes a novel experimental methodology to characterize the adhesion between two individual PLA filaments fabricated by FDM. Particular attention is devoted to the practical challenges of this type of testing, notably the difficulty of applying tensile loads exclusively through the top and bottom surfaces of the joint, as well as the curvature of the fracture surface and the complex state of stress. These limitations, and their potential impact on the interpretation of the results, are carefully examined to provide a critical and transparent assessment of the proposed method’s validity and reliability.

In summary, this study pursues four objectives:

1. Design of a specimen and fixturing system: to design a cruciform specimen incorporating a single PLA–PLA interlayer joint, together with a rapid, reliable, and reproducible clamping protocol suitable for high-throughput testing.
2. Characterize the mechanical properties of the single joint and its failure modes: to quantify the mechanical response of the isolated joint and to classify its failure modes under tensile loading (e.g., joint-controlled vs. filament-controlled).
3. Analyze the effect of the nozzle temperature (NT) and bed temperature (BT) on both the joint’s mechanical properties (apparent tensile strength) and the dominant failure mechanism.
4. Additional findings and methodological scope, to document and critically interpret any additional observations of interest, clarifying the validity, limitations, and applicability of the proposed method.

## 2. Materials and Methods

### 2.1. Materials

The material investigated in this study was polylactic acid (PLA, NatureWorks LLC, Minneapolis, Minnesota, USA) grade 4043D from Nature Works, supplied as filament spools with a nominal diameter of 1.75 ± 0.02 mm by Prusa Research (Prague, Czech Republic). This material has been reported to have a molecular weight (M_w_) of 110,000 g/mol to 120,000 g/mol and a d-lactide content of 5–6% [43]. These values are noteworthy because molecular weights higher than 50,000 g/mol provide stable mechanical properties, and the high d-lactide content hinders PLA crystallization during the FDM process [44].

### 2.2. Design of Samples and Clamps

Cross-shaped (cruciform) samples were designed with a specific geometry to determine the tensile strength for breaking an isolated PLA–PLA joint produced by FDM, together with a clamp system to attach the samples to the tensile test machine. The samples and the clamps were designed using FreeCAD software (v1.0) and sliced in PrusaSlicer software (v2.8.0). Two different clamping methods were analyzed. The first clamping designs were based on adhesives; the second designs were prepared for mechanical clamping. A reflection on the difficulties of each method is provided.

#### 2.2.1. Final Sample Design

**Figure 1:**
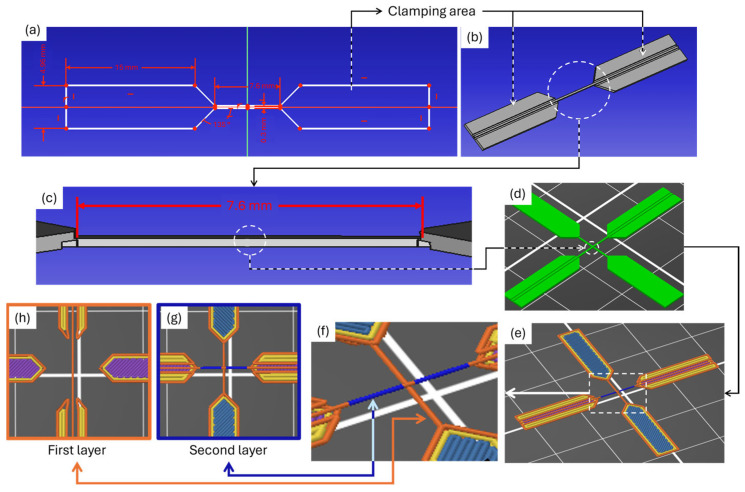
Design and slice of the cross-shaped samples: (**a**) sketch design of half of the sample where white lines are the sketch and red lines are the dimensions and constraints; (**b**) extruded sketch to form the solid object; (**c**) detail of the filament in which the joint is created; (**d**) final cross-shaped sample created in PrusaSlicer; (**e**) sliced sample; (**f**) detail of the joint; (**g**) second layer; (**h**) first layer.

First, half of the cross-shaped sample was sketched using FreeCAD software, as shown in Figure 1a. This design was extruded 0.45 mm in the *z*-axis to form the solid object with two large clamping areas, as shown in Figure 1b. Note that these clamping areas are necessary for mechanically clamping the samples into the tensile test device. Between these two clamping areas, a filament with a length of 7.6 mm is located, where the single joint is to be created, as shown in Figure 1c. This design was imported into the PrusaSlicer software, duplicated, and then rotated 180 degrees in the *x*-axis and 90 degrees in the *z*-axis. It was positioned in place to form a centred filament joint, as shown in Figure 1d. With this, the final design of the sample was obtained. This final design was sliced in PrusaSlicer to ensure the attainment of a two-layer structure, as shown in Figure 1e–h, over one hour. We refer to this testing method as the cruciform single-joint test (CSJT).

#### 2.2.2. Clamp Designs for Mechanical Test Fixturing

To enable mechanical testing of the cruciform specimens, bespoke clamps were designed to accommodate the cruciform’s geometries. Several clamping concepts were devised, fabricated using fused deposition modelling (FDM) in PLA, trialled, and iteratively refined until a configuration was obtained that permitted rapid, repeatable, and reliable tests.

**Figure 2:**
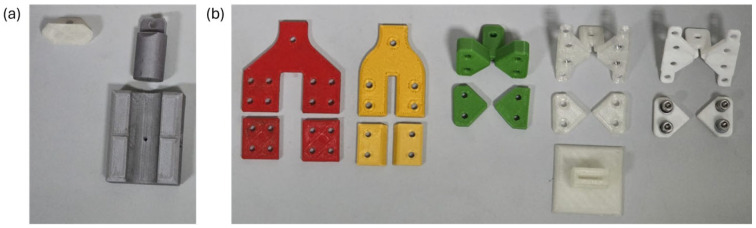
Example of some of the different clamps designed and FDM manufactured in PLA. Each group has its own clamping strategy: (**a**) adhesive clamping; (**b**) mechanical clamping. Different colors were used to visually reinforce that different versions of clamps were studied.

Two clamping mechanisms were explored: adhesive bonding to flat platens (Figure 2a) and mechanical clamping (Figure 2b).

For the adhesive route, paired flat platens were designed so that the cruciform specimen could be bonded to each face before loading. A comparative screening of adhesives (cyanoacrylate (Loctite SuperGlue-3), epoxy resin (Araldite Standard and Araldite Ultra), and polyurethane (Loctite PL Premium)) was conducted, drawing on prior experience within the group. Selection criteria emphasized turnaround time, bond integrity under tensile loading, and robustness against premature peel or shear at the specimen–platen interface.

For the mechanical route, both horizontal and vertical clamp architectures were evaluated. After preliminary trials, the horizontal mechanical clamp was adopted for all subsequent tests due to its operational simplicity, reusability, and improved control of alignment. The final design, its assembly, and its operating procedure are described in detail below.

### 2.3. FDM Sample Production

#### 2.3.1. FDM Printer

The FDM printer used was a Prusa model i3 MK3S+ (Prusa Research, Prague, Czech Republic), equipped with a 0.40 mm nozzle and a spring-steel build plate featuring a polyetherimide (PEI) surface coated with 3D LAC spray. The printer was operated in a laboratory room maintained at 22 ± 2 °C.

To improve thermal accuracy, the bed temperature was calibrated by measuring the surface temperature with a TOPDON TC001 thermal camera (Topdon Technology Co., Ltd., Shenzhen, China) using TCView software, and establishing a correlation between the actual surface temperature and the nominal set points reported by the printer firmware.

#### 2.3.2. First-Layer Calibration

**Figure 3:**
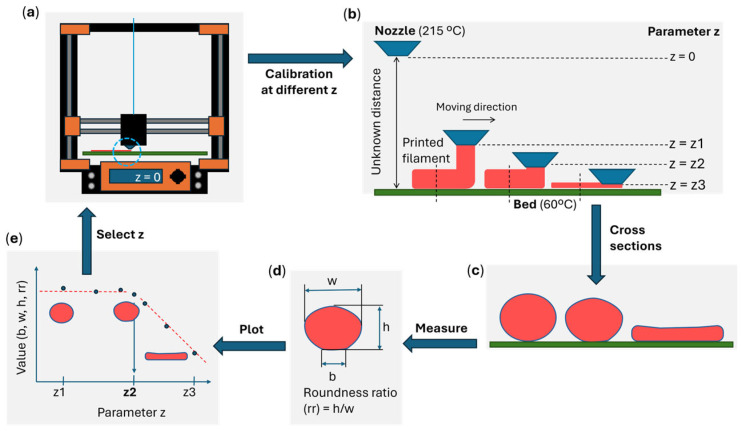
Scheme of first-layer calibration: (**a**) printer; (**b**) outcomes for different values of parameter z; (**c**) cross-section of filaments; (**d**) measured values on the cross-section (b, w, h) and calculated roundness ratio (rr); (**e**) plots of geometrical values (b, w, h or rr).

For the first-layer calibration, the in-built software of the printer was used, slightly adjusting the z parameter (giving values of z1, z2, …, z8) at nozzle temperature (NT) = 215 °C and bed temperature (BT) = 60 °C, as shown in Figure 3a,b. Cross-sections of these first-layer filaments were obtained by cutting frozen filaments (−20 °C) with a sharp blade and analyzed with the optical microscope NIKON SMZ800, (Nikon Corporation, Tokyo, Japan) under magnifications from ×10 to ×63, and ImageJ software (version 1.54p). In these images, the following measurements were taken: the base width (b), the maximum width (w), the maximum height (h), and the roundness ratio (rr, calculated as h/w), as shown in Figure 3d. After studying the different geometrical values for the parameter z, as shown in Figure 3e, and performing exploratory mechanical tests on samples printed under those conditions, the most precise yet useful value was selected for printing all the samples. Note that the desired separation between the bed and the nozzle is not the typical one used for obtaining a first layer, as later discussed in the results.

#### 2.3.3. Printing Samples and Storage

**Figure 4:**
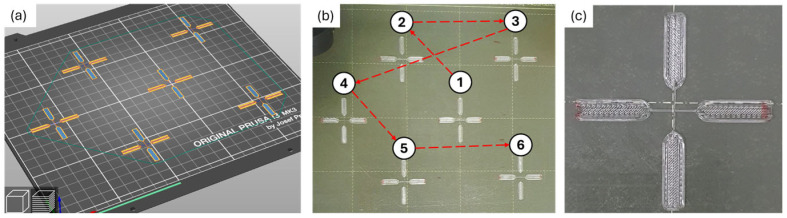
Printed samples. (**a**) Six samples printed in each run; (**b**) pictures of the six printed samples with the printing order indicated by the number 1 to 6; and (**c**) detail of a printed sample.

Samples were printed in runs, in which a total of six cross-shaped samples were printed per run, see Figure 4a,b. The first layer was printed directly on the bed, and the second layer was printed at a layer height increased by 0.20 mm. Printing speed was set to 35 mm/s for the filaments forming the joints. Different nozzle temperatures (NT = 180, 190, 200, 210, and 215 °C) and bed temperatures (BT = 30, 45, 60, 75, 90, 105, and 120 °C) were set. The reason for these temperatures is explained in the following subsection. Each sample was printed entirely before the following sample was printed, until all six samples per run were produced, requiring a total of nine minutes to print the six samples. After printing, all samples were marked with a red pen on the clamping area to keep track of the layer that was printed above, as shown in Figure 4c. All samples were allowed to cool down for a minimum of ten minutes on top of the bed before being manually removed with the help of a spatula. Printed samples were stored in a desiccator chamber to maintain low-humidity conditions (below 30% RH). This chamber was placed inside a dark room with no ambient light and with a controlled temperature of 22 ± 2 °C. The samples were naturally aged in these conditions for one day before testing.

#### 2.3.4. Deciding Printing Temperatures

A preliminary combination of nozzle temperature (NT) and bed temperature (BT) was chosen at 180 °C and 60 °C, respectively, after initial testing with different combinations of NT/BT (215/60 °C, 190/60 °C, and 180/60 °C). This combination was selected because it featured a mix of the two failure mechanisms (joint-controlled and filament-controlled) and was used for the first validation of the reproducibility of the designed samples and clamps, testing *n* = 60 samples on the final designs. The remaining printing temperature combinations (NT and BT) were determined iteratively.

The temperature iteration was performed by printing one combination of temperatures (starting from the lowest possible temperatures, NT = 180 °C and BT = 30 °C), testing the mechanical strength of a total of *n* = 12 joints for each combination, analyzing the results, and deciding on the following combination of temperatures until an end-of-interest condition was found. This end-of-interest condition was defined as the combination of temperatures (NT and BT) in which there were no detachments of the joints from the 12 samples tested, resulting in a pure filament-controlled failure region. Thus, it was expected that increasing either the NT or BT would lead to another end-of-interest condition. After the end-of-interest condition was found for a given NT and BT, the NT was increased by 10 °C, and the BT was rerun, starting from 30 °C and rising by 15 °C until the following end-of-interest condition was reached and until no more temperatures were available within the printer’s capabilities (the maximum possible BT was 120 °C).

### 2.4. Characterization of Printed Samples

#### 2.4.1. Optical and Fractographical Characterization

Printed samples were observed with the optical NIKON SMZ800 microscope. This has been used to analyze the morphology of the filaments and identify any geometry that could be of interest for improving the comparison of results with future researchers. In some of the samples, the filament printed on the bed (the one in the first layer) was cut with the aid of a sharp blade to determine the roundness ratio of the filament before freezing the samples at −20 °C.

#### 2.4.2. Thermal Characterization

**Figure 5:**
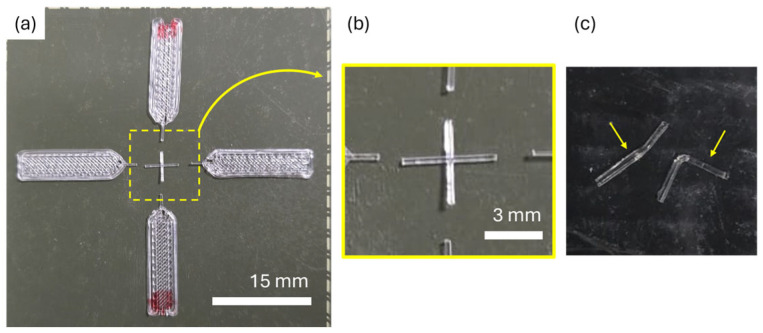
DSC sample: (**a**) printed sample; (**b**) detail of the material used for the DSC; (**c**) separated filaments from the same joint to ensure good contact with the bottom of the Al crucible, with small yellow arrows indicating the two separated filaments.

Differential scanning calorimetry (DSC) was performed on a Mettler Toledo 822e (Mettler Toledo, Columbus, OH, USA) using the STAResoftware (v11.00) at a heating rate of 10 °C/min on samples with a mass of 0.6 to 0.9 mg inside a 40 µL aluminum crucible. The small amount of material is explained as it was only analyzed, specifically the material corresponding to the joint and ~2 mm of the filament on each side adjacent to the joint, excluding the clamping areas, see Figure 5. The samples analyzed were those printed in position 1 (see Figure 4 for more details about the printing positions), as they were the most affected by the thermal conditions (longest residence time over the hot bed).

#### 2.4.3. Mechanical Characterization

**Figure 6:**
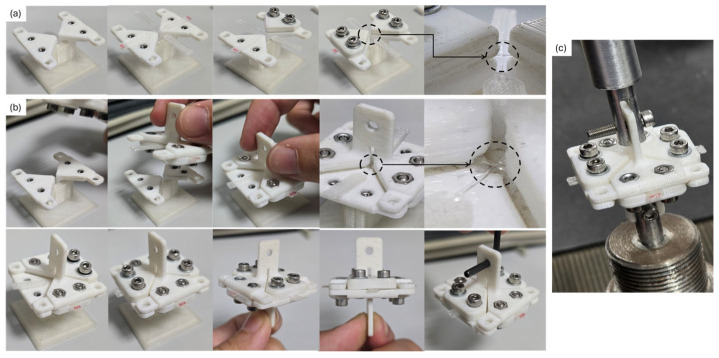
Mechanical clamping: (**a**) clamping of the second layer; (**b**) clamping of the first layer; (**c**) picture of the sample, mechanically clamped, prepared to be tested. Dashed boxes indicate detailed areas of the joint.

Tensile tests were conducted on an Instron 5866 universal testing machine (Instron, MA, USA), equipped with a 20 N load cell, to maximize force resolution. Tests were performed at a crosshead speed of 10 mm/min on specimens aged for 1 day under controlled laboratory conditions, in accordance with our established protocols for isolated 1D PLA filaments [27,28,29], as well as ISO 527-1:2019 [46] and ISO 291:2008 [47]. All specimens were fixed using horizontal mechanical clamps. A step-by-step pictorial guide to the fixturing procedure for each specimen is provided in Figure 6.

A minimum of *n* = 12 samples were tested for each combination of printing temperatures (NT and BT) using the horizontal mechanical clamping method. Special attention was given to the combination of NT = 180 °C with BT = 60 °C, where a total of *n* = 60 tests were performed to evaluate the effect of the printing position (positions 1 to 6) on the bed and assess the reproducibility of the designs.

All fractured specimens were classified into two categories according to their failure mechanism:

- Type 1: joint-controlled failure.
- Type 2: filament-controlled failure.

This classification was subsequently used to correlate the failure mechanisms with the printing temperatures.

**Figure 7:**
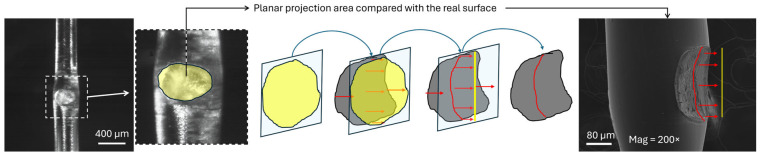
Planar projection compared to the real area. A comparison between an optical microscope image (plain) and an SEM image is shown. In this work, the planar area has been utilized. Grey area represents the SEM curved fracture, blue shield represents the projection plane, red arrows indicate the projection direction of the grey area into the blue plane, and yellow area represents the projection of the grey surface on the blue plane.

All samples were observed under an optical microscope before and after the mechanical test. Due to the difficulty of performing a uniaxial tensile test and properly characterizing the fracture surface and stress state, a simplified metric is proposed for this initial approach to evaluate mechanical strength: the apparent tensile strength (ATS), as shown in Equation (1).

(1) $$ATS=\frac{F_{max}}{A_{P}}$$

where $F_{max}$ is the maximum force obtained during the tensile test, and $A_{P}$ is the projected area (the planar projection of the fracture surface). This equation was only applied to the samples that failed due to joint detachment. To obtain the values of the projected area, the fracture surfaces of all samples were observed under an optical microscope at magnification 63×. A digital image was obtained from which the planar area of the fracture surface was measured using ImageJ software (Figure 7).

#### 2.4.4. Fractographical Characterization

Fractography of the detached joints was conducted on a ZEISS AURIGA FE-SEM (Carl Zeiss, Oberkochen, Germany). Before imaging, specimens received a ~15 nm carbon coating, deposited using a Leica carbon coater (Leica Microsystems, Wetzlar, Germany), to minimize electrical charging. Samples were mounted on copper plates using conductive carbon adhesive tape and examined at an accelerating voltage of 3 kV, a working distance of 16.3 mm, and a detector of SE/BSE.

## 3. Results

### 3.1. Bed Temperature Characterization

Thermal characterization of the bed surface revealed that the measured temperatures were consistently lower than the nominal values reported by the printer, which were above 60 °C. Because the surface temperature exhibited spatial non-uniformity, both the minimum and maximum values were recorded and used to compute an area-averaged bed temperature for each set point. For clarity, we distinguish between nominal (software-reported) and real (measured) temperatures. The corresponding nominal–real temperature pairs, together with the associated ranges, are summarized in Table 1.

### 3.2. Calibration of Parameter z

**Figure 8:**
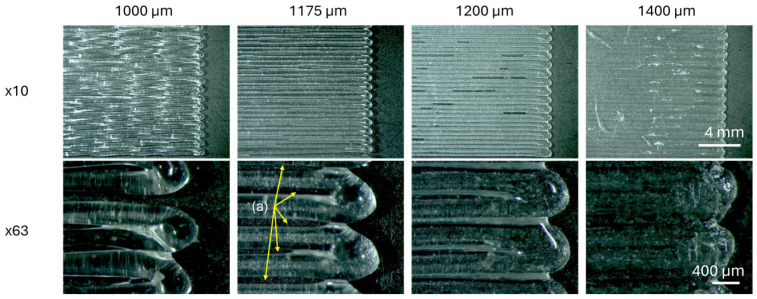
Top views of the first layers printed with different parameter z values. Images captured using an optical microscope at various magnifications. (a) Gaps between filaments in the first layer.

For the first-layer calibration, it is typically desired that the distance between the nozzle and the bed produces a compact first layer with minimal gaps between filaments. In Figure 8, examples of the first layers produced for different parameter z values are shown. From these images, note that a typical first layer would be printed at a parameter z of −1400 µm, as it has no gaps between the filaments.

**Figure 9:**
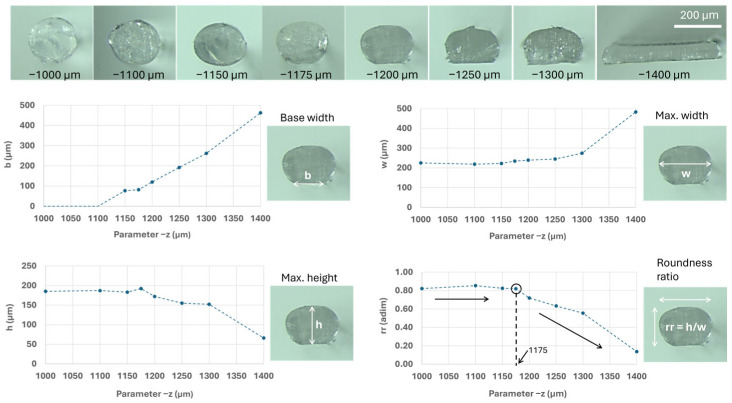
Cross-section views of first-layer calibration samples obtained with an optical microscope under ×63 magnification. Measured geometries are plotted against the parameter z, seeking a clear definition for the first layer in the threshold of the roundness ratio with z = −1175 µm. Dashed lines joining the dots are used for better visualization of the trends. Black circle and dashed line indicated the chosen value for z. Black large arrows indicate rr trends.

However, for this research, it was preferable to use the −1175 µm, although the first-layer calibration prints exhibited gaps. This is explained in relation to the cross-section images of single filaments printed over the bed, where their dimensions (b, w, h, and rr) are plotted against the parameter z in Figure 9, and also due to the positive results from the exploratory mechanical tests performed at 180/60 °C (later presented).

It was found that the value of −1175 µm was the optimal one for printing the samples, as the roundness ratio presented a relatively straightforward threshold, with stable values around 0.82, and a decrease after the parameter z values reached −1175 µm, as shown in Figure 9. After confirming through mechanical tests that the samples were detaching at the joint (a mandatory requirement for the design) and that this condition provided a mix of failure mechanisms (ideal for serving as a reference point), the value of −1175 µm was selected for this study. Note that each printer will have its own calibration method, and it is recommended to compare the results with the optical images of the cross-sections in Figure 9. This should help reproduce and compare results.

### 3.3. Optical Characterization of Printed Samples

**Figure 10:**
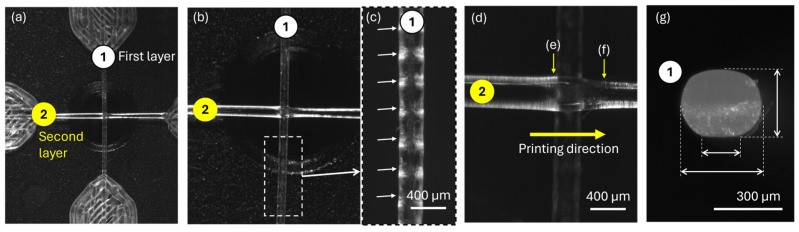
Optical images of a cross-shaped sample at NT = 180 °C and BT = 60 °C. (**a**) Under 10× magnification, numbers inside circles are indicating first and second layer; (**b**) under 30× magnification, dashed box indicating detailed area in next subfigure; (**c**) detail of wavy surface in layer 1, note the homogenously distributed valleys indicated with arrows; (**d**) under 63× magnification, showing details of the joint; (**e**) accumulation of material before to the joint; (**f**) decrease of material after the joint; (**g**) roundness ratio measured on a samples first layer, with a value of 0.87 for the sample in the image.

Once the parameter z was defined at –1175 µm, cross-shaped samples were printed at NT = 180 °C and BT = 60 °C. The samples were carefully analyzed with an optical microscope, see Figure 10, highlighting the following results: (1) A wavy surface on the sample printed over the bed, see Figure 10b,c. This is related to the step motor used during extrusion, which produces slight micro-over- and under-extrusions. As previously reported by our group, this wavy morphology does not affect the mechanical properties of the filament [45]. (2) An accumulation of material in front of the joint, see Figure 10d,e. This is explained as the second layer of the filament, which is deposited, finding the filament already deposited on the bed and accumulating the PLA filament in front of it. (3) A decrease in section is found after the joint, see Figure 10d,f, as the flow of material has been diffused by the first-layer filament. (4) The roundness ratio calculated in the filaments printed in the bed was consistent with the values obtained during the first-layer calibration, with values between 0.77 and 0.87.

No significant influence of the printing temperatures (neither nozzle nor bed) was observed on these geometries. Higher nozzle temperatures (NT = 250 °C) did produce significant variations in the filaments, but they are outside the scope of the results presented here, as they fall in the end-of-interest regions.

### 3.4. Clamping Methods

**Figure 11:**
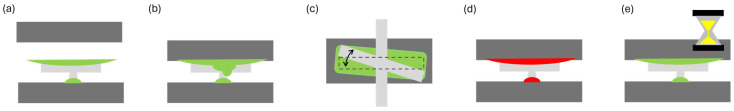
Scheme of issues found using adhesive clamps (dark grey for the clamp and green for the adhesive) for testing the cruciform samples (light grey): (**a**) adhesion failure; (**b**) excess of adhesive that flows into the joint; (**c**) rotation of filaments in the clamps before the adhesive sets, were the dashed box represents the desired position and the arrow indicates its displacement; (**d**) heating of the adhesive (red color used to indicate that the adhesive is hot); and (**e**) excessive curing times for being practical.

Our initial clamping strategy relied on the adhesive bonding of the cruciform specimens to flat support plates. However, this approach did not yield a reliable or rapid test workflow. In practice, it suffered from the following limitations, schematized in Figure 10:

1. Interface debonding: specimens frequently detached at the specimen–platen interface rather than failing at the intended interlayer bond, Figure 11a.
2. Adhesive flow: capillary action causes the adhesive to flow into the joint region, locally reinforcing the joint, as shown in Figure 11b.
3. Geometric distortion: differential adhesion to the support plates introduces twist and eccentricity, promoting bending and parasitic stresses in the joint (Figure 11c).
4. Heating: specific formulations exhibited exothermic curing reactions, altering the thermal history of the printed polymer prior to testing (Figure 11d).
5. Time penalties: epoxy and PU-based adhesives used required extended curing times to achieve adequate bond strength, thereby undermining the throughput objective of the method (Figure 11e).
6. Clamp reusability: Each clamp had to be discarded after each test. It required printing many clamps, resulting in a subsequent time delay and increased material and energy consumption.

Given these constraints, adhesive clamping was found to be unsuitable for fast, reliable, and reproducible testing. Due to these considerations, designs in which clamping is performed by mechanical means were ultimately chosen as the approach to carry out an extensive characterization of the samples, as it was found to be fast, more reliable, and reproducible. Moreover, the clamps could be reused and clamping each sample required just 1 min. Regarding the vertical or horizontal approach, the horizontal one was found to be faster and more reliable, as the samples can be printed flat on the bed. With this method, it takes approximately 20 min to produce six samples. These six samples can be tested in under 30 min. What takes longer is the 1-day storage time. However, if that storage time was not used, it could be possible to obtain information about the properties of the joint in just 1 h for *n* = 6, and no more than 2 h for *n* = 12, considering printing, testing, and analyzing. Of course, the 3D printer must be well-calibrated, but that is a process inherent to any machine used in research.

The vertical clamping method required printing the clamps vertically against the bed (just by twisting 90 degrees toward the clamping area in the design and adding supports), which added extra layers to the sample and the need for supports to the design, making it more time-consuming, less material efficient, and more prone to fail during the print and with minor defects through the joint filaments.

The main drawback of the final chosen mechanical clamping method is that the test is not a pure tensile test, and the stress state becomes more complex. As a first approach, a simplified mechanical indicator, known as apparent tensile strength (ATS), was proposed for comparing the mechanical performance of the detached samples. The ATS is a simplified metric for evaluating the results in a first approach and evaluate the potential of the CSJT. Future work supported in FEM will be used for a deeper understanding of stress state and the correlation between the ATS and the real mechanical properties of the joint.

### 3.5. Tensile Test Failure Types

**Figure 12:**
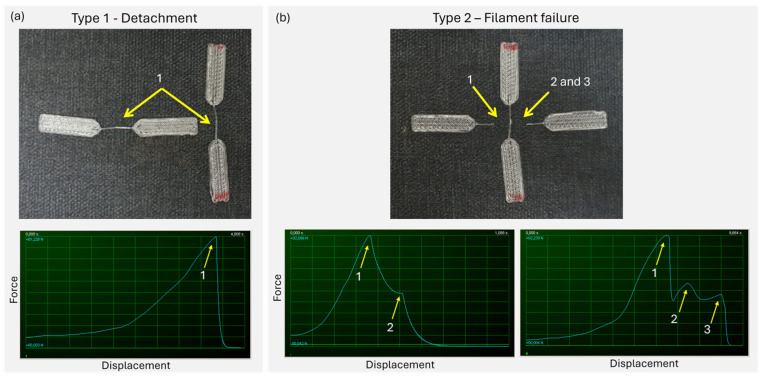
Failure types: (**a**) type 1, failure due to detachment of the joints with arrows indicating the only failure point; example of a force-displacement curve obtained from the test, with a yellow arrow indicating the only one peak of force corresponding to the whole detachment of the joint; (**b**) type 2, failure due to failure of the filaments next to the joint with arrows indicating the two failure points next to the joint; example of force-displacement curve obtained for type 2 failure, were two peaks of force are observed, attributed to two fragile fractures of the filaments adjacent to the joint; example of a force displacement obtained for type 2 failure, were three peaks of force are observed, the first corresponding to a fracture of one side the filament adjacent to the joint, the second point corresponding to the necking of the other side of the filament still attached to the joint and the third point corresponding to the final fracture of the filament adjacent to the joint. Small text in the force-displacement curves is not relevant, but the number of force peaks.

After determining the clamping method for this work, as well as the final design and printing conditions of the samples, a total of more than 800 tests were performed on the cross-shaped samples. During these mechanical tests, two types of failure were observed: detachment of the joint (type 1 failure) and filament failure (type 2 failure). These two failure modes are shown in Figure 11. In the type 1 failure, it was observed that the joint failed, resulting in the detachment of the filaments with only one failure point, the joint, as shown in Figure 12a. However, in the type 2 failure, it was found that the failure of the adjacent filaments next to the joint occurred, with two fracture points, as shown in Figure 12b. The difference between type 1 and type 2 failures was easily identified in the force–deformation curves directly obtained during the tensile test: type 1 failures exhibited a single peak of force, whereas type 2 showed two or more peaks.

Every tested sample was categorized into one of these two groups (type 1 or type 2) based on the type of failure presented. With this distribution in two groups, the probability of a joint presenting a type 1 failure under the different tested conditions was calculated (detachment ratio). The error probability has been assumed to be ±1 broken sample. Later, the ATS was calculated with the results from the detached samples.

### 3.6. Effect of Printing Position

**Figure 13:**
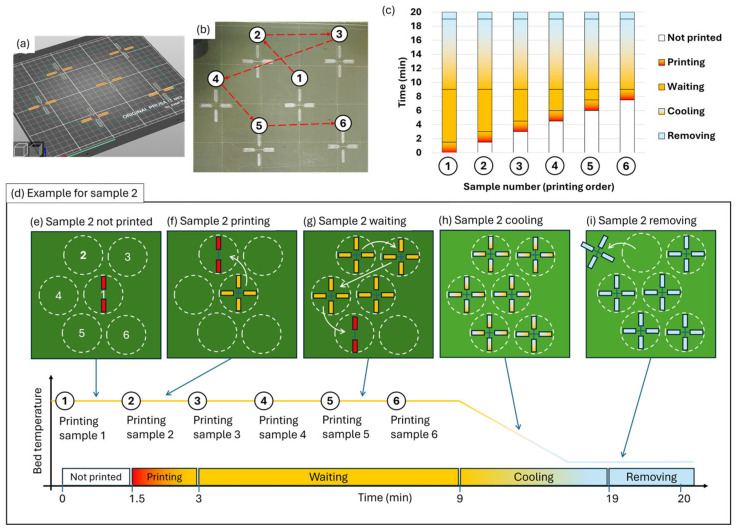
Printing positions and residence time over the bed for the six samples printed in each run: (**a**) slicer; (**b**) printed samples; (**c**) scheme of the printing process times for each sample; (**d**) scheme of the printing process step by step; (**e**) printing first sample in position 1 from the total of 6 positions over the bed, each position represented with a dashed circle and number, were sample two is not printed yet; (**f**) printing second sample, the printer starts producing a complete cruciform sample in position 2; (**g**) second sample is waiting over the hot bed, with arrows indicating the consecutive production of samples in position 3, 4 and 5; (**h**) all samples printed in their corresponding position and cooling for a minimum of 10 minutes over the bed; (**i**) removing the samples, were it is shown sample two being removed from the bed.

In this study, samples were printed in batches of six samples per print, each sample in a different position over the bed. As samples are printed over a hot bed surface, and the first sample printed remains over the hot bed until the last sample is printed, there is the possibility that the first sample significantly differs in properties from the last printed sample. To evaluate the effect of the printing temperatures and time spent over the hot bed surface, differential scanning calorimetry (DSC) was performed on the first sample printed (position 1), as it remained on the hot plate for a longer time at a higher temperature. It was performed to determine whether all the samples printed in the same run can be considered similar or not. A scheme of the six printed samples and their positions is presented in Figure 13.

In this scheme, it is possible to observe that the sample printed in position 1 remained longer over the hot bed (Figure 13e–i), justifying the focus on position 1 samples to assess the maximum effect of the printing temperatures. A deeper analysis of the effect of the hot bed on the samples printed is out of the scope of this research. Special attention was given to the sample first printed at 180/120 °C, which is closer to the PLA crystallization temperatures, as well as to the reference condition of 180/60 °C. Crystallinity values obtained from the DSC tests are provided in Table 2.

Notably, the table reveals that the two conditions analyzed in more detail, with *n* = 3 samples each, yielded the same average value and standard deviation.

**Figure 14:**
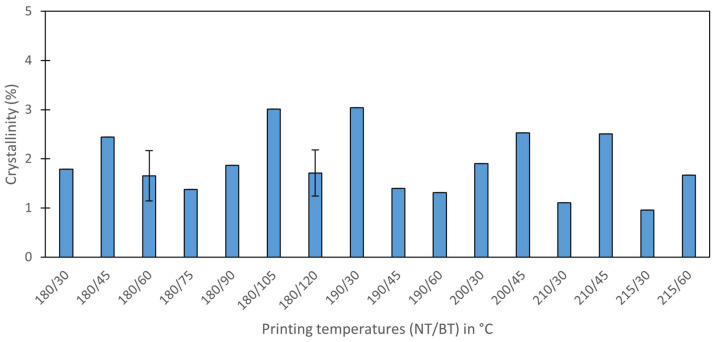
Crystallinity values of samples printed in position 1, the samples that remained the longest time in the hot bed.

For a clearer view of the results, crystallinity values have been plotted against the different printing temperatures (Figure 14). It can be observed that the crystallinity differences are negligible, with no clear trend evident with the printing temperatures. With this, a deeper study on samples printed on other positions (shorter time over the hot bed) is discarded.

Regarding the samples that remained on top of the bed at a nominal 120 °C, they were expected to be in the crystallization temperature regime (the crystallization of α’ starts at 80 °C [48]). However, the DSCs revealed that even these samples with the highest expected crystallinity had values smaller than 3%. This can be explained with regard to the following: (1) the highest measured bed temperature was 111 °C, smaller than the nominal bed temperature (120 °C), (2) the high d-lactide content (5 to 6%) of the PLA studied highly decreases its crystallization kinetics [44], (3) the small contact area between the joint and the bed minimizes the energy transferred to the joint, (4) the thermal gradient through the sample as PLA is a bad thermal conductor, and (5) the energy is lost to the surroundings, especially since the second-layer filament is not in contact with the bed, but floating over it. Although direct measurements of the actual temperature of the printed PLA were not performed, it can be observed that the actual temperatures were significantly lower than the nominal 120 °C, with several mechanisms further decreasing it, associated with a decrease in crystallization kinetics. Regarding previous studies that correlated the influence of crystallinity with mechanical properties, the maximum effect of this crystallinity on the tensile strength should be approximately 3% [49]. Crystallinity values between samples are even smaller, being sufficiently small to consider any difference negligible [49,50,51] in all printing conditions. It demonstrates that the effect of just 9 min of difference between samples printed in position 1 (first printed) and position 6 (last printed) is not significant, supporting the analysis of all samples together as if they were printed in the same position.

**Figure 15:**
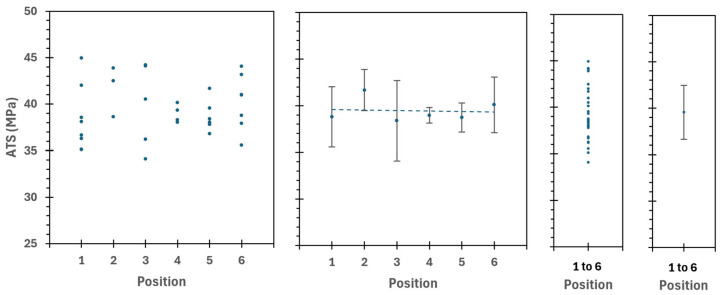
ATS vs. position 1 to 6 calculated in all samples 180/60 °C that detached. Samples in position 1 remained approximately nine minutes more over the hot bed compared to samples printed in position 6. Dashed lines shows the linear fitting, no trend is observed with the printing position (also referred as printing order and related with the residence time over the bed).

To further support these results, a total of *n* = 60 tests for samples printed at NT = 180 °C and BT = 60 °C were performed. They were used to confirm the reproducibility of the tests with the mechanical clamps and to evaluate the effect of the printing position (time over the hot bed). It was found that there was no significant effect of the printing position on the bed (and residence time on the bed) in the ATS. All the ATS values calculated for the samples 180/60 °C are plotted against the position on the bed in Figure 15.

It can be observed that there is no significant difference among all the samples analyzed, not even when comparing the samples with the most considerable difference during processing (position 1 vs. position 6), which is approximately nine minutes in residence time over the bed. Even more, the average ATS calculated with the data of all samples is similar to the linear regression line that fits to the individual average value for each position. The temperatures below the crystallization temperature, as well as the low heat accumulated during the printing process (due to the sample’s shape and the setup used), and especially the high d-lactide content in the PLA used, are consistent with no differences between these samples. All these tests support the reproducibility of the designed experiment and the similitude of the six samples printed per run.

### 3.7. Effect of Printing Temperature

Drawing on an extensive experimental campaign comprising over 800 individual tests across all specimen iterations, this report details the most pertinent subset to the thermal-processing study: 246 tensile tests specifically designed to investigate the influence of nozzle and bed temperatures on single-joint performance. This large sample size shows the repeatability and reliability of the derived metrics (detachment ratio and ATS), thereby enabling confident discrimination of temperature effects and failure regimes. A summary of the results for each temperature condition is provided in Table 3.

**Figure 16:**
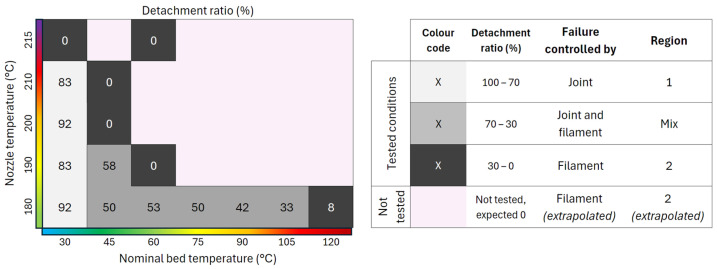
Heat map of detachment ratio for different printing temperatures showing the various regions regarding the predominant failure mechanism: joint-controlled failure, filament-controlled failure, or a mix of both.

Using the complete set of tests presented in Table 3, along with the corresponding detachment ratios, a heat map was constructed to visualize the percentage of specimens exhibiting joint detachment for each nozzle–bed temperature combination (Figure 16). The map delineates three regions according to detachment prevalence:

- Region 1 (joint-controlled): detachment ratio ≥ 70%.
- Mixed region: detachment ratio 30–70%, indicating coexistence of joint- and filament-controlled failures.
- Region 2 (filament-controlled): detachment ratio ≤ 30%. Extrapolation of the trends observed was used to extend this region.

This representation provides a compact processing–failure map, clarifying how temperature controls the dominant failure mechanism and offers a practical guide to parameter selection when targeting either interfacial assessment (high detachment prevalence) or filament-dominated response (low detachment prevalence).

The nozzle temperature had a sharp threshold, where a 5 °C increase from 210 °C to 215 °C decreased the detachment ratio from values around 80–90% to 0%. On the other hand, bed temperature produced a more gradual decrease in the detachment ratio, being stable from 45 °C to 90 °C.

**Figure 17:**
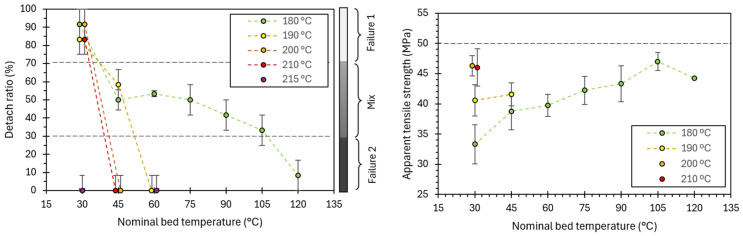
Effect of printing temperature and bed temperature on the detachment ratio and the apparent tensile strength (ATS). Only samples that detached were used for calculating the ATS. Back dashed lines are solely used for improving data visualization by directly joining the points.

From the subset of specimens that failed by joint detachment (failure type 1), the apparent joint tensile strength, ATS, was computed and analyzed to quantify the influence of printing temperatures. Restricting the analysis to Type-1 failures avoids mixing distinct failure regimes. The resulting ATS values, as a function of nozzle and bed temperature, are presented in Figure 17.

The pronounced reduction in detachment above ~210 °C suggests two things: (1) a threshold for which interdiffusion of polymeric chains becomes sufficient, enabling bond coalescence beyond a critical entanglement, and (2) the regime failure of this bond has a low dependency on the residence time over the hot bed, at least, for low bed temperatures (30 °C). In contrast, it is observed that BT primarily modulates cooling rates, resulting in a smoother gradient in both detachment probability and ATS. It is consistent with the 3D-printed samples tested in the literature, where it is described that increasing the printing temperature improves interlayer adhesion [23,24,31,32,33].

Regarding the apparent tensile strengths, it is observed that a slight increase in the nozzle temperature has a higher impact on the strength than the bed temperature. It leads to the conclusion that the nozzle temperature is the primary energy used to form the bonding between the two filaments. On the other hand, BT has a lower effect, by just reducing the cooling rate of the filaments and allowing the filaments to bond during a more extended period at the necessary high temperatures prompted by the NT. This interesting result, in which it is observed that the NT is predominant for bond formation, is made possible by studying the isolated joint.

Interestingly, there is a limit to the maximum apparent tensile strength (ATS) at approximately 49–50 MPa in these cross-shaped samples. This value lies below the mechanical strength of the bulk PLA, which for PLA4043D stabilizes at 62–65 MPa [43], suggesting that the limit is governed more by specimen geometry and boundary conditions than by the intrinsic strength of the polymer. In particular, (1) the clamping system which is fixed next to but not directly on top of the joint, obtaining a biaxial stress state, (2) the curvature of the weld between filaments, which also promotes a more complex stress state, and (3) the use of a projected, nominal cross-section to compute the ATS, whose correlation to the real tensile strength of the joint is still to be determined.

**Figure 18:**
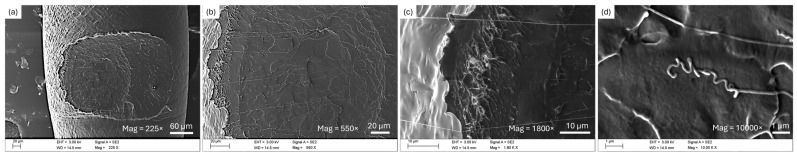
Detail of the plastic deformation in the fracture surface. Image taken on the upper filaments (second layer) of a sample printed at NT = 180 °C and BT = 60 °C showing (**a**) top view, (**b**) detail of the inner fracture, (**c**) detail of the edge, (**d**) detail of a plastic nanofilament.

SEM images obtained from broken samples (Figure 18) show plastic deformation on the borders of the joint, as well as throughout the entire inner fracture. Notice how, in Figure 18b,c, the joint borders are aligned with the tensile loads of the test, perpendicular to the fracture surface, indicating their deformation during the test. Additionally, in Figure 18d, a highly plasticized polymeric nanofilament is depicted. These plasticized filaments were found throughout the entire fracture surface. This is consistent with a good adhesion between the two filaments in the weld, with adhesion strong enough to produce stresses beyond the plastic limit before detaching.

**Figure 19:**
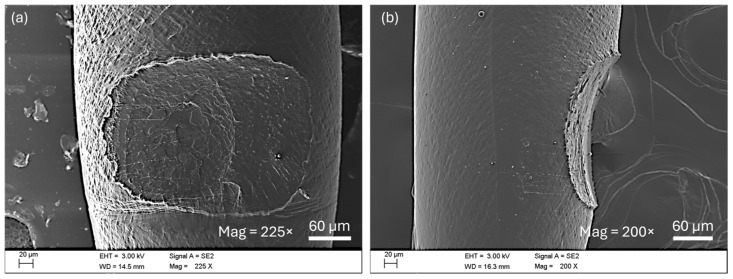
Detail of fracture surface on the upper filaments (second layer) of a sample printed at NT = 180 °C and BT = 60 °C. SEM images showing (**a**) top view and (**b**) lateral view.

In Figure 19, SEM images of the same samples are presented from two different viewpoints, clearly showing a curved fracture surface. This image is provided to remind us that the detailed analysis of the fracture surface shape and the precise calculation of the stress state for determining the bonding strength are left for future research.

### 3.8. Effect of Storage Time

To further understand the results presented, the effect of storage time (or ageing time) is evaluated. Regarding the storage time, a total of 288 tests were evaluated to assess the influence of natural ageing on the PLA–PLA joint strength. Table 4 provides the results obtained. Tests performed at 1 day of ageing are like those previously analyzed.

**Figure 20:**
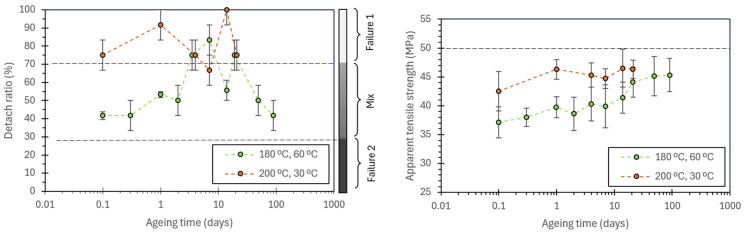
Effect of natural ageing time on detach ratio and apparent tensile strength. Samples studied were printed under two conditions: with a nozzle temperature of 180 °C and a nominal bed temperature of 60 °C (reference), and with a nozzle temperature of 200 °C and a nominal bed temperature of 30 °C to confirm the trend. Dashed lines are solely used for improving data visualization by directly joining the points.

In the table, it is observed that the evolution has been analyzed with the ageing time of the samples printed in the reference temperatures 180/60 °C, but also in the 200/30 °C temperatures. The combination of 200/30 °C was selected as it had mechanical values close to the 50 MPa limit and it was evaluated if this; apparently, stabilised sample (from the ATS point of view at 1 day) remained stable or evolved over the 50 MPa limit. For better visualization, the results are plotted in Figure 20.

The detachment ratio remained similar, with no significant effect of natural ageing. Samples printed at 180/60 °C had a mix of failure types 1 and 2, whilst the sample printed at 200/30 °C remained with a predominant failure type 1 (detachment).

In samples printed at 180/60 °C, there is a significant increase in the mechanical properties from samples aged 0.1 days to 90 days. This increase is consistent with the expected results from natural ageing in low-humidity conditions at room temperature [45]. Although the simplified ATS metric had a stabilised value of 50 MPa, this value might be related to the stabilised tensile strength of 62 MPa obtained in pure tensile tests in single filaments printed by FDM. This correlation between the ATS and the real mechanical properties is left for future work.

Samples printed at 200/30 °C showed no significant increase in mechanical properties with ageing time, indicating that they were more aged once printed. Future research should aim to understand the distinct thermal histories of the printed samples, thereby facilitating a more comprehensive discussion of these results.

An additional set of 192 tests were performed on samples aged 14 days for all temperature combinations to obtain an overview of the effect of ageing. Table 5 presents the results of samples aged 14 days.

**Figure 21:**
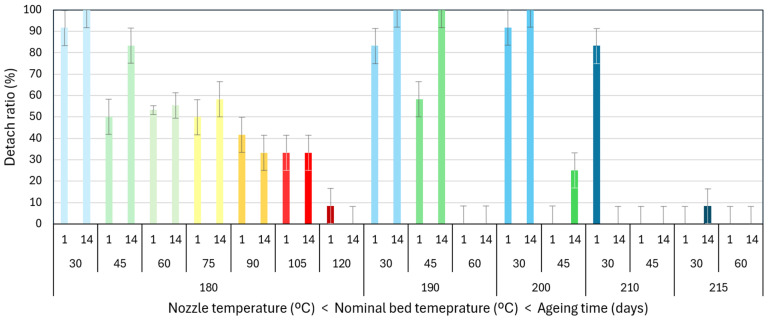
Detach ratio comparison between samples aged 1 day and 14 days. For all printing temperatures, tested at 10 mm/min. Colors are just to help the visualization of the different conditions. Blues are for BT = 30 °C, greens for BT = 45 °C and 60 °C, and so on. Darker color has been used to indicate higher nozzle temperatures.

**Figure 22:**
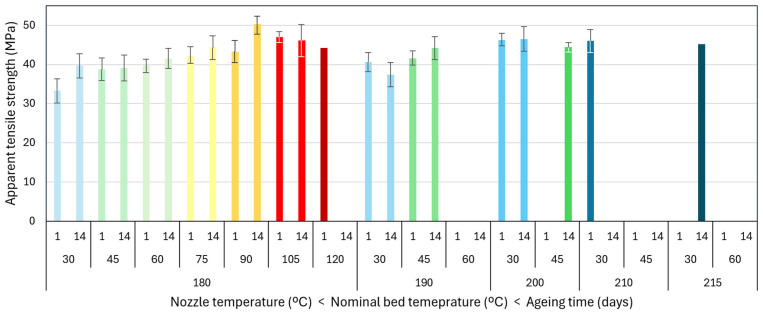
Apparent tensile strength comparison between samples aged 1 day and 14 days. For all printing temperatures, tested at 10 mm/min. Colors are just to help the visualization of the different conditions. Blues are for BT = 30 °C, greens for BT = 45 °C and 60 °C, and so on. Darker color has been used to indicate higher nozzle temperatures.

Figure 21 and Figure 22 compare the results of samples aged 14 days with the previously discussed results from 1 day of ageing.

Comparing samples aged 1 day with samples aged 14 days, it is noted that for samples printed at a nominal bed temperature of 45 °C, the detach ratio significantly increases. Samples 210/30 °C also showed a significant decrease in the detachment ratio. The reason behind these variations remains unclear. It justifies the need to explore the characterization of materials in simplified scenarios. This will help us to understand the fundamental mechanism underlying the variation in material properties.

Regarding the mechanical properties, there is a significant increase in samples 180/30 °C and 180/90 °C. The remaining samples do not exhibit a notable change. Still, none of the samples provided an average ATS value higher than 50 MPa.

### 3.9. Effect of Crosshead Speed

Additionally, the effect of the crosshead speed during the tensile tests has been evaluated to provide a better contextualization of the results obtained. A total of 156 tests were evaluated to assess the influence of crosshead speed on these PLA–PLA joints. Samples were printed at 180 °C and 60 °C. The crosshead speed is given in mm/min and not normalized as min^−1^, as it was unclear how to normalize it with respect to the sample length. Table 6 provides the results obtained. Tests performed at 10 mm/min are common with those previously analyzed.

**Figure 23:**
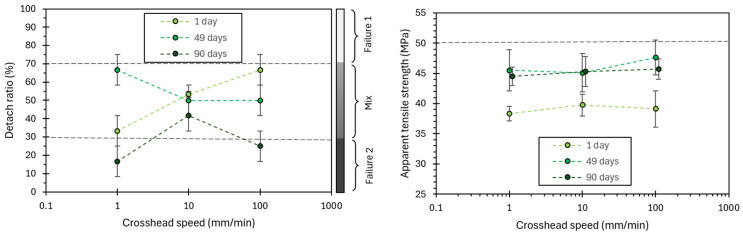
Effect of crosshead speed (mm/min) on the detach ratio and apparent tensile strength. Colors are just to help the visualization of the different conditions. Dashed lines are solely used for improving data visualization by directly joining the points.

For a better visualization of the results, these results are plotted in Figure 23.

About the effect of the crosshead speed on the detachment ratio, no correlation is observed. The observed variations remain to be understood. Regarding the effect on mechanical strength, it is found that the crosshead speeds studied have no significant impact on the ATS. This is true for samples aged 1 day, 49 days, and 90 days, indicating that these samples may be more aged than the single PLA filaments described in the literature [43,44,45]. This could be consistent with the cruciform samples having a longer residence time at higher temperatures and slower cooling rates over the bed. It is also consistent with the smaller effect of the storage time previously discussed and with literature results that correlate that aged samples are less influenced by the crosshead speed [43]. However, the design of the experiment may affect it as well, as it is not a pure tensile test. Extending to faster speeds could be of interest for simulating PLA joints under non-quasi-static conditions.

## 4. Conclusions

Based on over 800 tests, we developed and validated a novel minimal-material methodology to assess the apparent mechanical strength of a single joint between two FDM-printed PLA filaments, utilizing the simplest possible geometry. The principal conclusions are as follows:

1. Sample design and fixturing. A cruciform specimen incorporating a single filament–filament joint was designed and evaluated under alternative clamping strategies. A rapid mechanical clamp proved the most reliable and operationally straightforward approach for high-throughput testing. Nevertheless, the current geometry constrains difficult direct tensile loading through the joint, which should be addressed in future iterations to reduce parasitic bending stresses.
2. Failure mapping and strength metric. Cross-shaped specimens were printed, stored, and evaluated under controlled conditions, enabling a contextualized analysis of joint response. A detachment ratio (probability of detachment failure) was introduced to capture the binary nature of failure mechanisms (Type-1 vs. Type-2). For joints that detached, the apparent joint tensile strength, ATS, was computed from tensile data assisted by optical micrographs. This framework yielded a temperature–failure map that distinguishes joint-controlled from filament-controlled regimes.
3. Thermal-processing effects. Nozzle temperature (NT) and bed temperature (BT) exerted significant and distinct influences. Increasing NT and BT reduced detachment probability and raised ATS. The response to NT displayed a threshold-like transition, consistent with enhanced chain mobility and interdiffusion at the weld, requiring a minimum of energy for it to happen. In contrast, BT produced a smoother trend, plausibly due to decreased cooling rates, slightly increasing the time that the joint is at the required temperatures for the interdiffusion of polymeric chains in contact in the joint.
4. Upper bound in apparent tensile strength. A maximum value for the ATS was observed around 50 MPa. This plateau likely reflects that the limit is governed more by specimen geometry and boundary conditions than by the intrinsic strength of the polymer, which properties are closer to 62-65 MPa [43,44,45]. Within these boundaries, the ATS metrics remain coherent with the literature and was found useful for a first approach.

The authors are currently conducting a study on the effect of different variables (such as sample shape and nozzle diameter) to achieve a deeper understanding of the properties of a stable and reproducible FDM-printed joint. The authors are also currently extending the response metrics beyond the apparent tensile strength (ATS) to include interfacial fracture energy (G_c_) and interlaminar shear strength, in order to obtain more discriminating maps of joint performance. The results of this ongoing research will be published shortly.

Together, these developments aim to establish the cruciform single-joint test (CSJT) as a reliable and pioneering system for studying how processing variables govern interlayer bonding in FDM. Beyond offering a high-throughput, minimal-waste route to quantify joint performance, the methodology provides actionable inputs to process simulations and model calibration, thereby supporting more rational processing–structure–property maps for polymer AM that were not available until now.

## Figures and Tables

**Table 1:** Bed temperatures: the nominal temperature, the minimum and maximum temperatures measured, the calculated average temperature, and the difference between nominal and average temperatures. The error in measured temperatures is estimated to be ±0.5 °C.

Nominal (°C)	Minimum (°C)	Maximum (°C)	Average (°C)	Difference (°C)
30	29.0	30.6	29.8	−0.2
45	44.8	45.9	45.4	+0.4
60	55.4	58.4	56.9	−3.1
75	68.4	71.5	70.0	−5.0
90	81.7	83.1	82.4	−7.6
105	96.0	96.9	96.5	−8.5
120	109.6	112.3	111.0	−9.0

**Table 2:** DSC scan results. Samples from position 1, tested 30 to 60 min after printed.

Nozzle Temperature (°C)	Nominal Bed Temperature (°C)	Ageing Time (Days)	Position	Samples Tested (*n*)	Crystallinity (%)
180	30	0	1	1	1.8
180	45	0	1	1	2.4
180	60	0	1	3	1.7 ± 0.5
180	75	0	1	1	1.4
180	90	0	1	1	1.9
180	105	0	1	1	3.0
180	120	0	1	3	1.7 ± 0.5
190	30	0	1	1	3.1
190	45	0	1	1	1.4
190	60	0	1	1	1.3
200	30	0	1	1	1.9
200	45	0	1	1	2.5
210	30	0	1	1	1.1
210	45	0	1	1	2.5
215	30	0	1	1	1.0
215	60	0	1	1	1.6

**Table 3:** Test of samples printed at different temperatures. All samples were aged for 1 day and tested at a rate of 10 mm/min.

Nozzle Temperature (°C)	Nominal Bed Temperature (°C)	Ageing Time (Days)	Crosshead Speed (mm/min)	Samples Tested	Detachment Ratio	Apparent Tensile Strength (MPa)
180	30	1	10	12	0.92 ± 0.08	33 ± 3
180	45	1	10	18	0.50 ± 0.06	39 ± 3
180	60	1	10	60	0.53 ± 0.02	40 ± 3
180	75	1	10	12	0.50 ± 0.06	42 ± 2
180	90	1	10	12	0.42 ± 0.08	43 ± 3
180	105	1	10	12	0.33 ± 0.08	47 ± 2
180	120	1	10	12	0.08 ± 0.08	44 ± 2
190	30	1	10	12	0.83 ± 0.08	41 ± 3
190	45	1	10	12	0.58 ± 0.08	42 ± 2
190	60	1	10	12	0.00 + 0.08	-
200	30	1	10	12	0.92 ± 0.08	46 ± 2
200	45	1	10	12	0.00 + 0.08	-
210	30	1	10	12	0.83 ± 0.08	46 ± 3
210	45	1	10	12	0.00 + 0.08	-
215	30	1	10	12	0.00 + 0.08	-
215	60	1	10	12	0.00 + 0.08	-

**Table 4:** Test of samples naturally aged for printing temperatures of 180 °C/60 °C and 200 °C/30 °C. The error for the detach ratio was assumed to be ±1 detached sample. Error of the apparent tensile strength is calculated as the standard deviation.

Nozzle Temperature (°C)	Nominal Bed Temperature (°C)	Ageing Time (Days)	Crosshead Speed (mm/min)	Samples Tested	Detachment Ratio	Apparent Tensile Strength (MPa)
180	60	0.1	10	48	0.42 ± 0.02	37 ± 3
180	60	0.3	10	12	0.42 ± 0.08	38 ± 2
180	60	1	10	60	0.53 ± 0.02	40 ± 3
180	60	2	10	12	0.50 ± 0.08	39 ± 3
180	60	4	10	12	0.75 ± 0.08	40 ± 3
180	60	7	10	12	0.83 ± 0.08	40 ± 4
180	60	14	10	18	0.56 ± 0.06	41 ± 3
180	60	21	10	12	0.75 ± 0.08	44 ± 3
180	60	49	10	12	0.50 ± 0.08	45 ± 4
180	60	90	10	12	0.42 ± 0.08	45 ± 3
200	30	0.1	10	12	0.75 ± 0.08	43 ± 3
200	30	1	10	12	0.92 ± 0.08	46 ± 2
200	30	4	10	12	0.75 ± 0.08	45 ± 2
200	30	7	10	12	0.67 ± 0.08	45 ± 2
200	30	14	10	12	1.00 ± 0.08	47 ± 3
200	30	21	10	12	0.75 + 0.08	46 ± 2

**Table 5:** Additional tests were conducted on samples that had naturally aged for 14 days at all printing temperatures. The error for the detach ratio was assumed to be ±1 detached sample. Error of the apparent tensile strength is calculated as the standard deviation.

Nozzle Temperature (°C)	Nominal Bed Temperature (°C)	Ageing Time (Days)	Crosshead Speed (mm/min)	Samples Tested	Detach Ratio	Apparent Tensile Strength (MPa)
180	30	14	10	12	1.00 − 0.08	40 ± 3
180	45	14	10	12	0.83 ± 0.08	39 ± 3
180	60	14	10	18	0.56 ± 0.06	41 ± 3
180	75	14	10	12	0.58 ± 0.08	44 ± 3
180	90	14	10	12	0.33 ± 0.08	50 ± 2
180	105	14	10	12	0.33 ± 0.08	46 ± 4
180	120	14	10	12	0.00 + 0.08	-
190	30	14	10	12	1.00 − 0.08	37 ± 3
190	45	14	10	12	1.00 − 0.08	44 ± 3
190	60	14	10	12	0.00 + 0.08	-
200	30	14	10	12	1.00 ± 0.08	47 ± 3
200	45	14	10	12	0.25 ± 0.08	44 ± 1
210	30	14	10	12	0.00 + 0.08	-
210	45	14	10	12	0.00 + 0.08	-
215	30	14	10	12	0.08 ± 0.08	45
215	60	14	10	12	0.00 + 0.08	-

**Table 6:** Test of samples tested at different crosshead speeds. The error for the detach ratio was assumed to be ±1 detached sample. Error of the apparent tensile strength is calculated as the standard deviation.

Nozzle Temperature (°C)	Nominal Bed Temperature (°C)	Ageing Time (Days)	Crosshead Speed (mm/min)	Samples Tested	Detachment Ratio	Apparent Tensile Strength (MPa)
180	60	1	1	12	0.33 ± 0.08	38 ± 1
180	60	1	10	60	0.53 ± 0.02	40 ± 3
180	60	1	100	12	0.67 ± 0.08	39 ± 3
180	60	49	1	12	0.67 ± 0.08	46 ± 3
180	60	49	10	12	0.50 ± 0.08	45 ± 4
180	60	49	100	12	0.50 ± 0.08	48 ± 3
180	60	90	1	12	0.17 ± 0.08	45 ± 2
180	60	90	10	12	0.42 ± 0.08	45 ± 3
180	60	90	100	12	0.25 ± 0.08	46 ± 2

## Data Availability

The datasets presented in this article are not readily available because the data are part of an ongoing study. Requests to access the datasets should be directed to jaime.orellana@upm.es.
